# Supplementary material for: Evaluation of Novel Doxorubicin-Loaded Magnetic Wax Nanocomposite Vehicles as Cancer Combinatorial Therapy Agents
Source: Pharmaceutics. 2020 Jul 7;12(7):637. doi: 10.3390/pharmaceutics12070637 (PMC7407097; doi:10.3390/pharmaceutics12070637)
Supplement: Supplementary file 1 [file pharmaceutics-12-00637-s001.pdf]

pharmaceutics

Supplementary Materials: Evaluation of Novel Doxorubicin-Loaded Magnetic Wax Nanocomposite Vehicles as Cancer Combinatorial Therapy Agents

Julia Jiménez-López, Lorena García-Hevia, Consolación Melguizo, Jose Prados, Manuel Bañobre-López and Juan Gallo

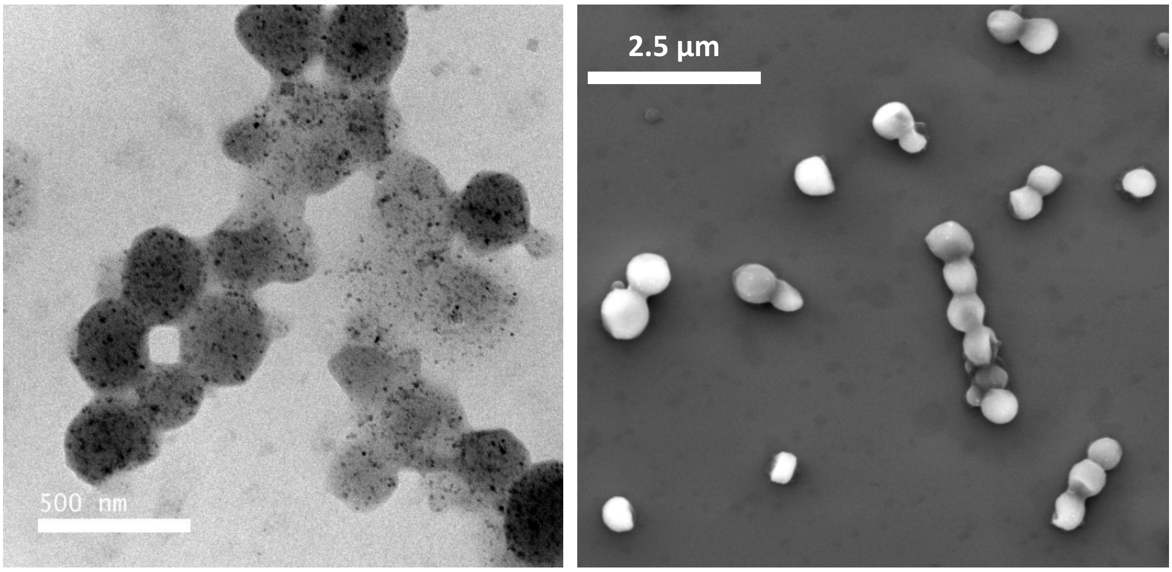

(**a**) (**b**)

Figure S1. (**a**) Overview TEM micrograph of mWNVs-DOX showing small hipointense spots coming from the iron oxide nanoparticles, inside larger wax structures. (**b**) Overview SEM micrograph of mWNVs-DOX.

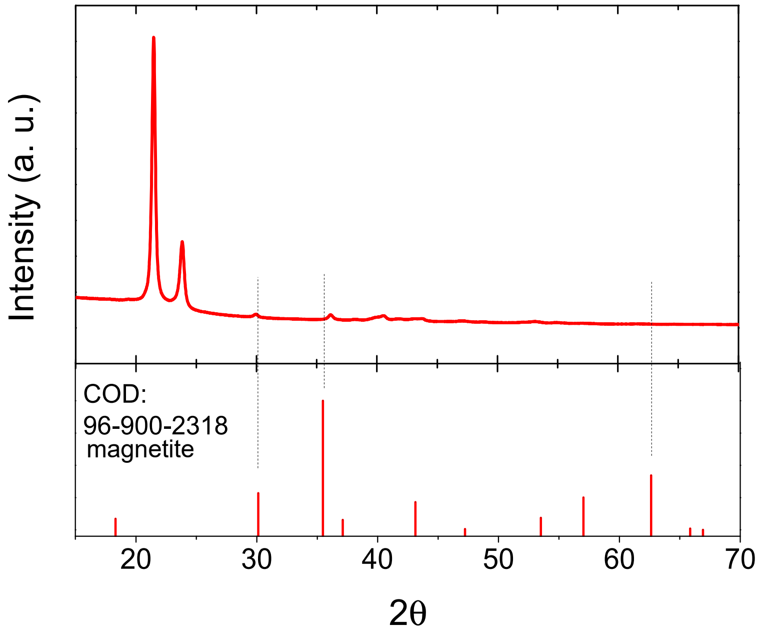

Figure S2. XRD pattern of mWNVs. The spectra is dominated by two peaks at low θ coming from the wax. Fe_3O_4 peaks can also be observed and match well COD 96-900-2318 Fe_3O_4 data.

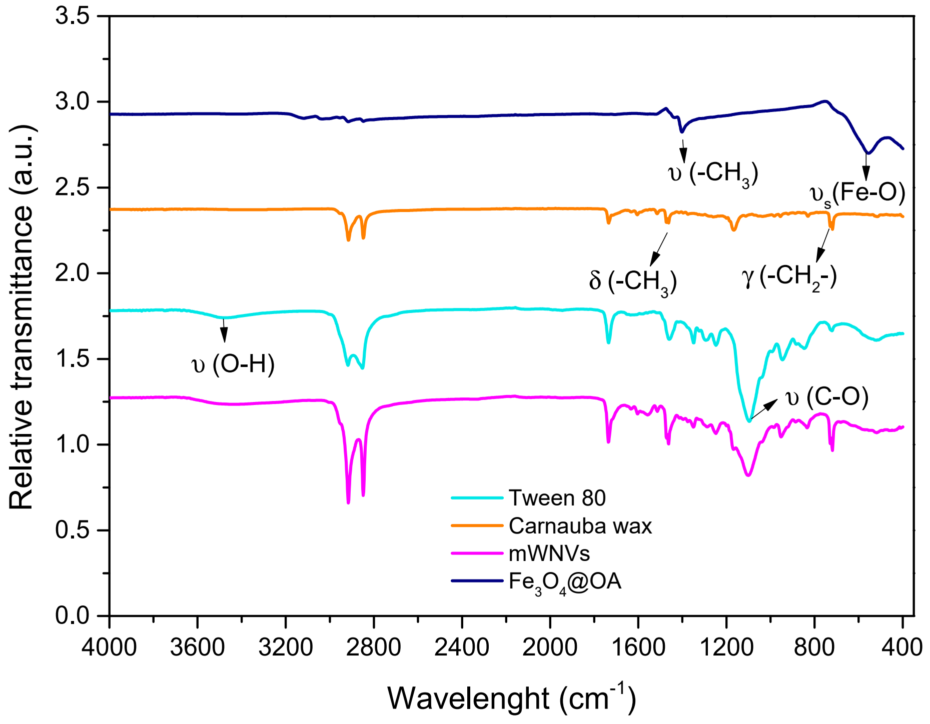

Figure S3. FTIR spectra of the final mWNVs and its components.

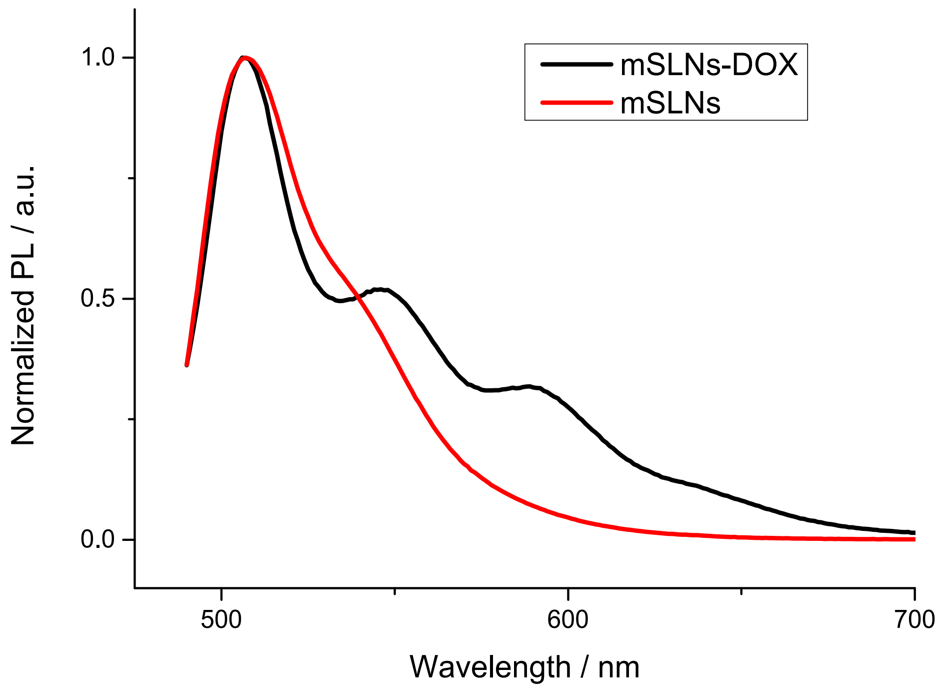

Figure S4. Fluorescence spectra of mWNVs and mWNVs-DOX (λ_{exc} = 470 nm) showing a main peak at 510 nm coming from the dye DiO and two weaker shoulder at higher wavelenghts coming from DOX.

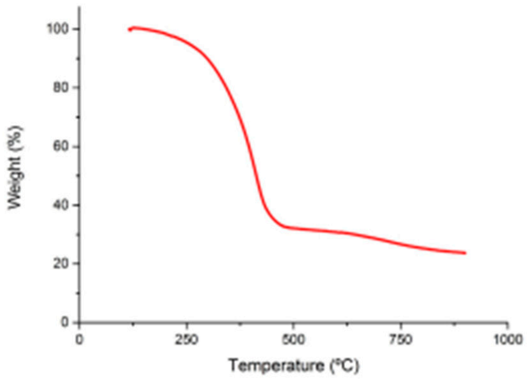

Figure S5. Thermogravimetric analysis of mWNVs showing a main decrease in mass around 350 °C coming from the decomposition of the wax. The final mass at 900 °C matches well the initial MNP loading.

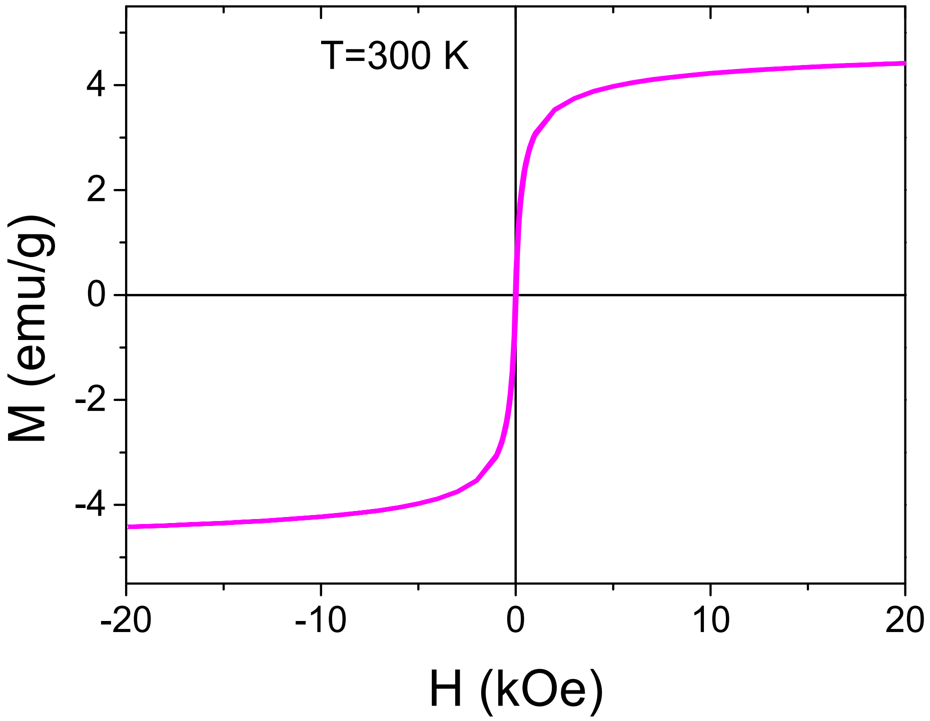

Figure S6. Hysteresis loops in the applied magnetic field range from −20 to +20 kOe at 300 K for the mWNVs-DOX formulations, showing a clear superparamagnetic behaviour.

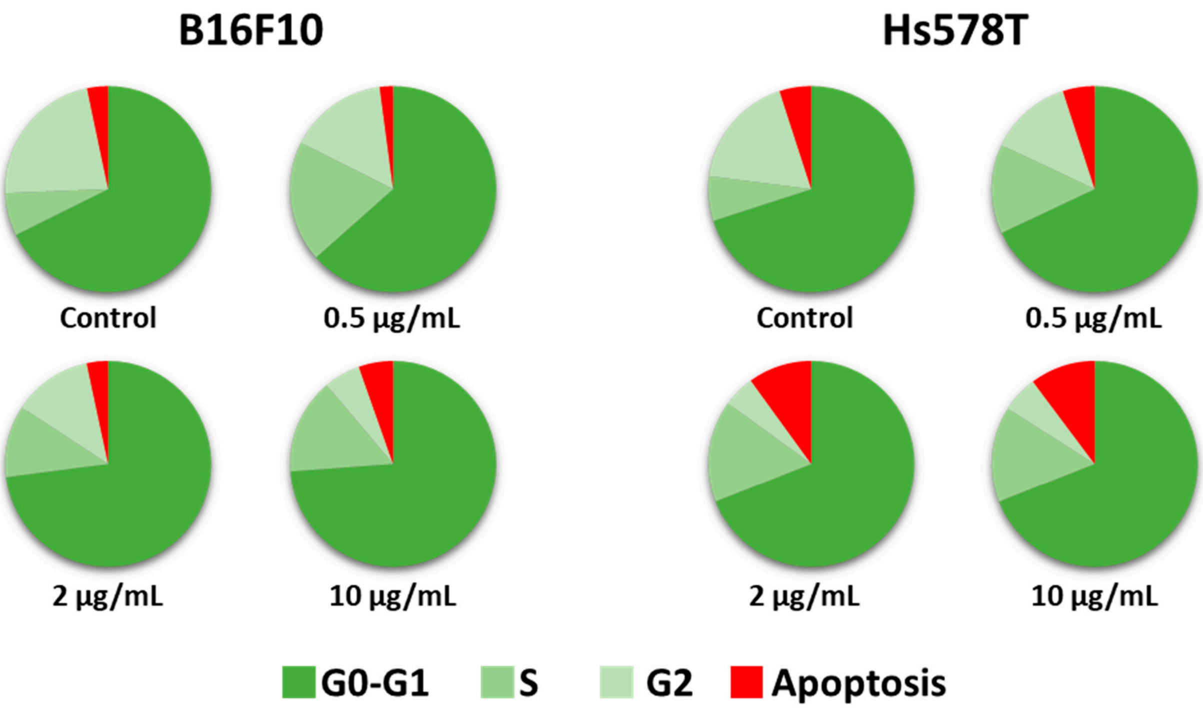

Figure S7. Flow cytometry evaluation of B16F10 and Hs578T cells in 2 D after 48 h of exposure to a wide range of control unloaded mWNVs concentrations (0.5–10 µg/mL).

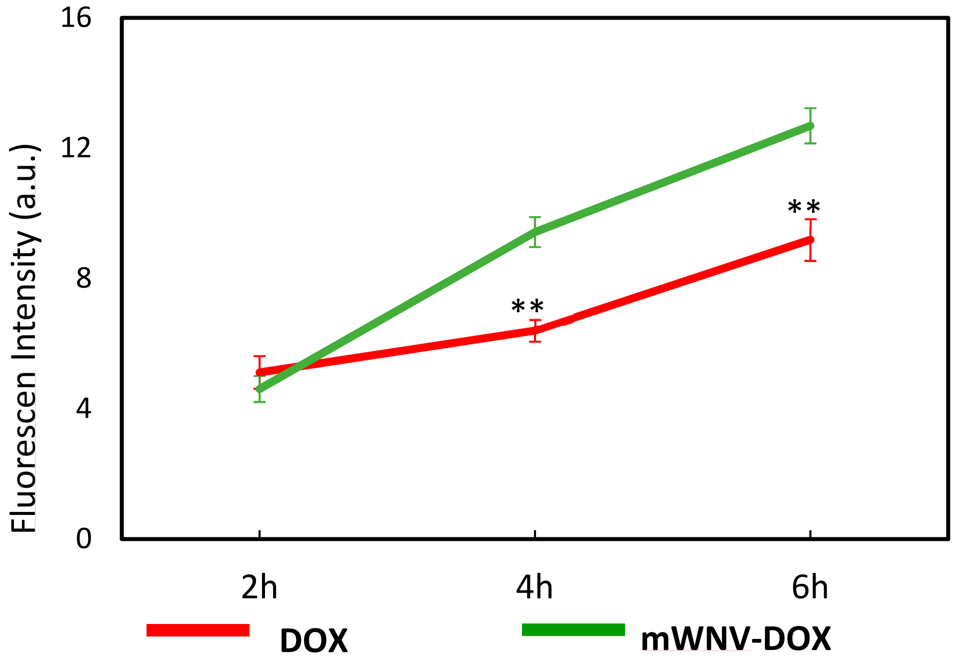

Figure S8. Intracellular DOX quantification from confocal images of B16F10 cells incubated with DOX (red) versus mWNV-DOX (green) at a concentration of 2 µg/mL for up to 6 h. ** $p < 0.005$, Student t test.

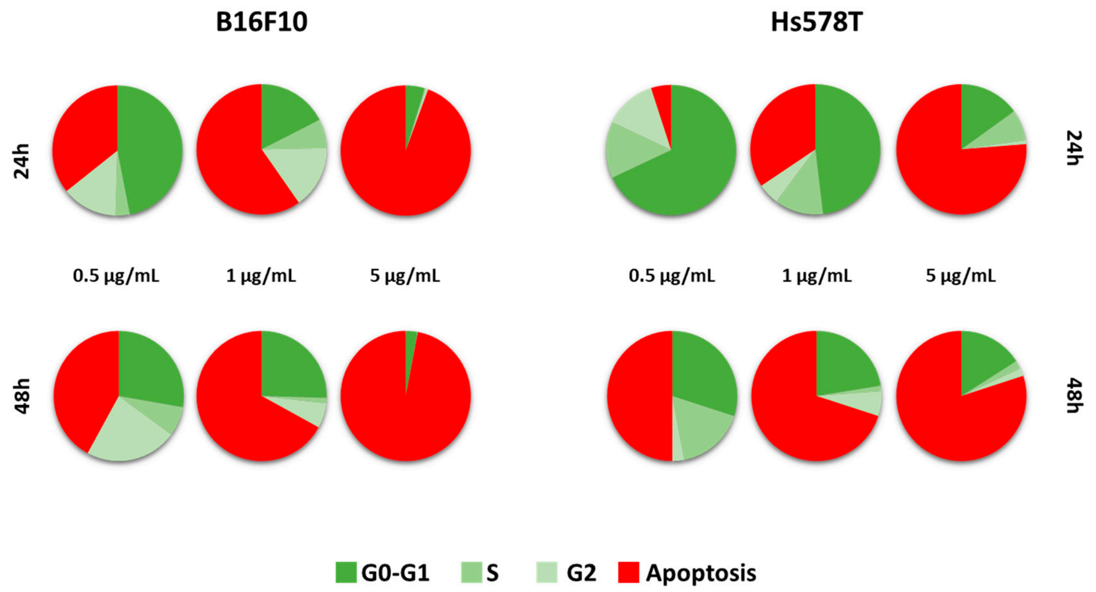

Figure S9. Flow cytometry evaluation of B16F10 and Hs578T cells in 2 D after 48 h of exposure to a wide range of mWNVs-DOX concentrations (0.5–10 µg/mL).

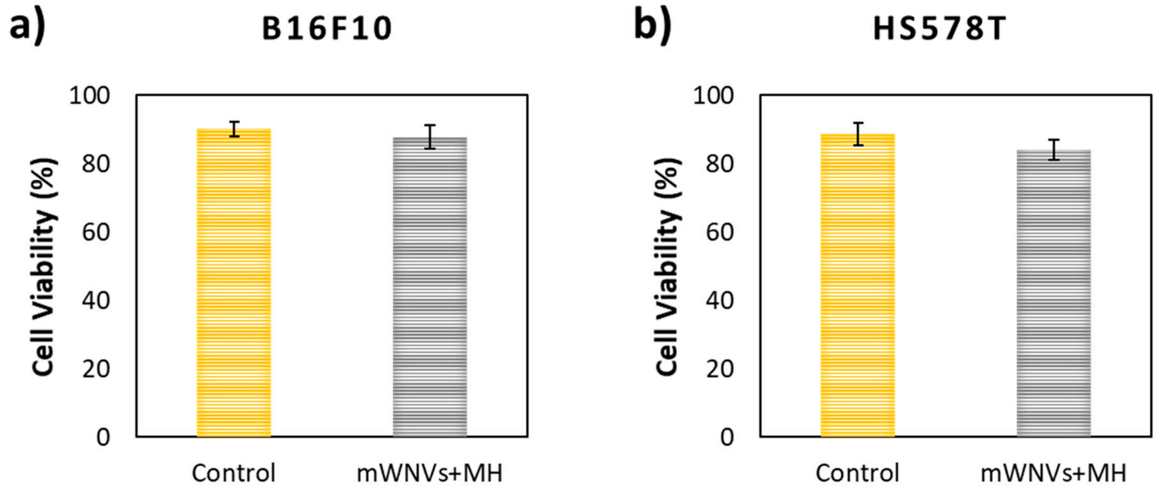

Figure S10. (**a**) Cell viability of B16F10 cells under control conditions (yellow, no mWNVs) and after exposure to 0.5 µg/mL of mWNV and 1h hyperthermia (grey). (**b**) Cell viability of Hs578T cells under control conditions (yellow, no mWNVs) and after exposure to 0.5 µg/mL of mWNV and 1h hyperthermia (grey). No statistical differences were observed.

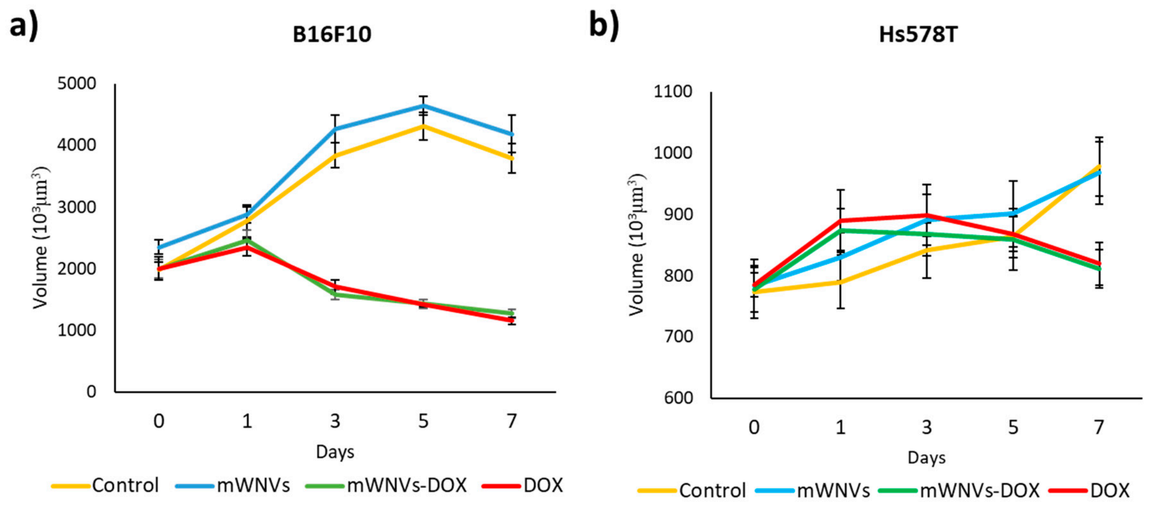

Figure S11. Volume evolution of (**a**) B16F10 MCTSs and (**b**) Hs578T MCTSs untreated (yellow) and treated with DOX (red), control unloaded mWNVs (blue), or mWNV-DOX (green) measured for 7 days.

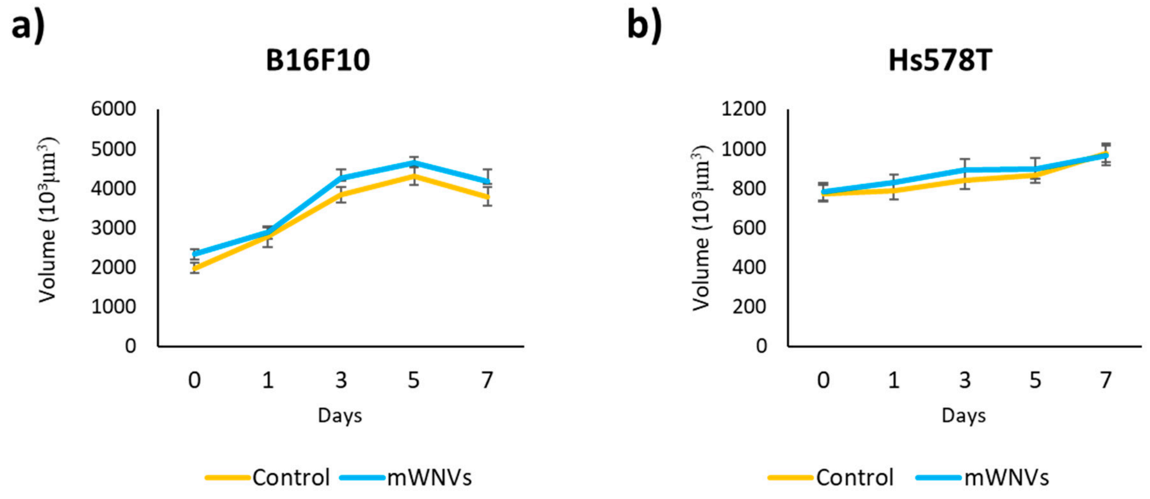

Figure S12. (**a**) Volume evolution of control B16F10 MCTSs (yellow) and B16F10 MCTSs treated with control unloaded mWNVs (blue). (**b**) Volume evolution of control Hs578T MCTSs (yellow) and Hs578T MCTSs treated with control unloaded mWNVs (blue).

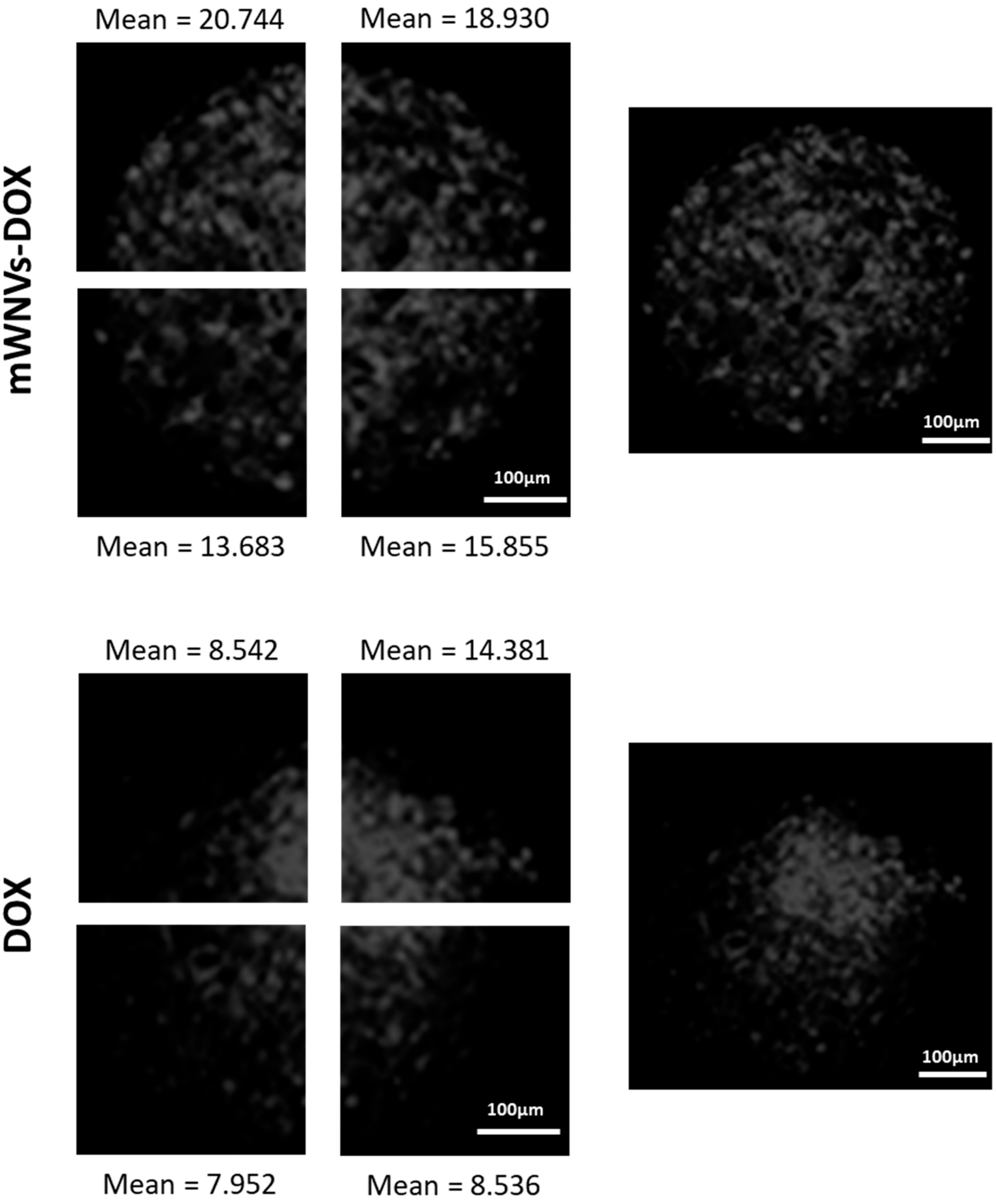

Figure S13. Fluoresce homogeneity analysis of LSM images showing DOX distribution in MCTSs treated with mWNVs-DOX (top) versus DOX (bottom).

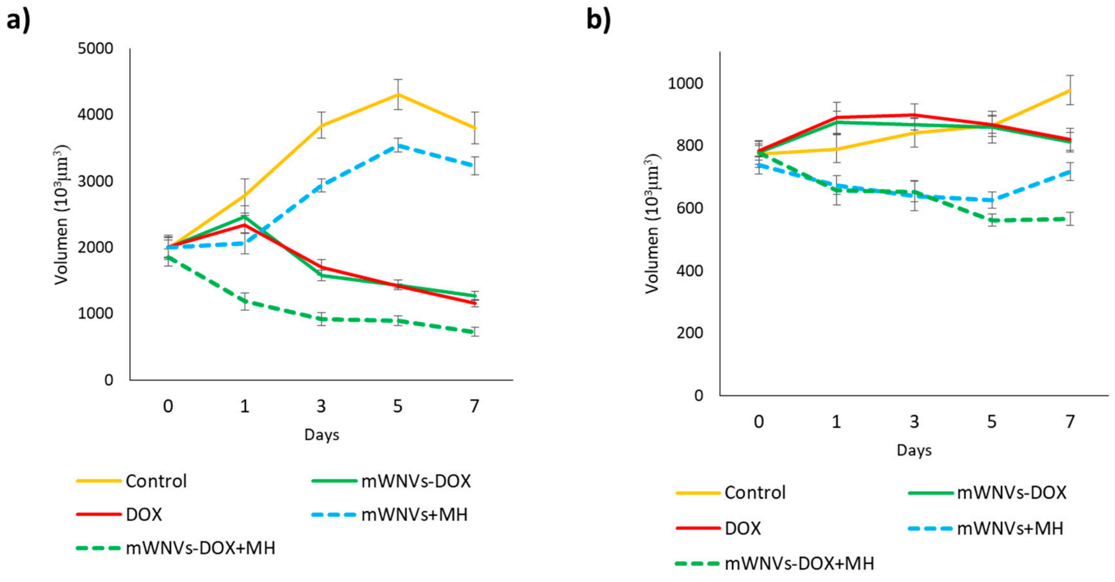

Figure S14. Volume evolution of (**a**) B16F10 MCTSs and (**b**) Hs578T MCTSs untreated (yellow) and treated with control unloaded mWNVs plus MH (blue), DOX (red), mWNV-DOX (green), and mWNV-DOX plus MH (green dotted) measured for 7 days.

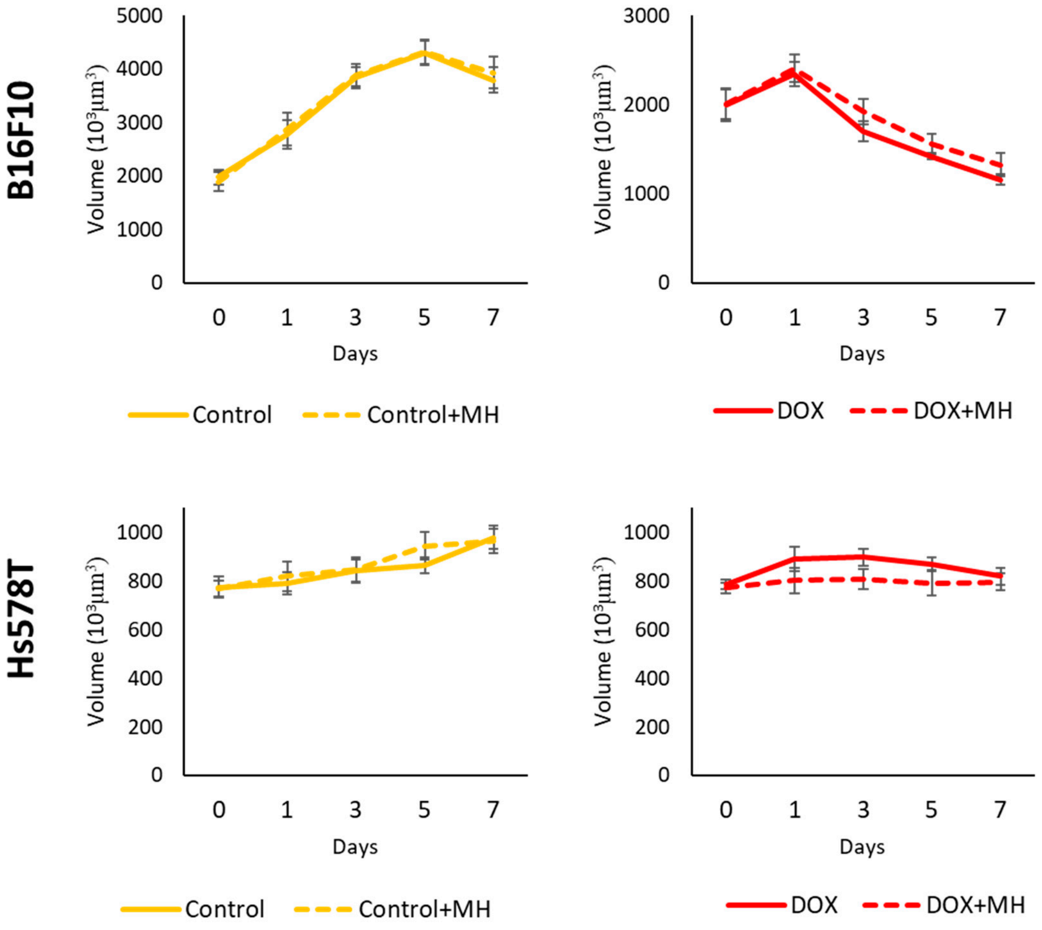

Figure S15. Volume evolution of B16F10 MCTSs (top) and Hs578T MCTSs (bottom) treated with control unloaded mWNVs (yellow) and DOX (red), alone (solid lines) and under 1h magnetic hyperthermia application (dotted lines) measured for 7 days (mean ± SEM).

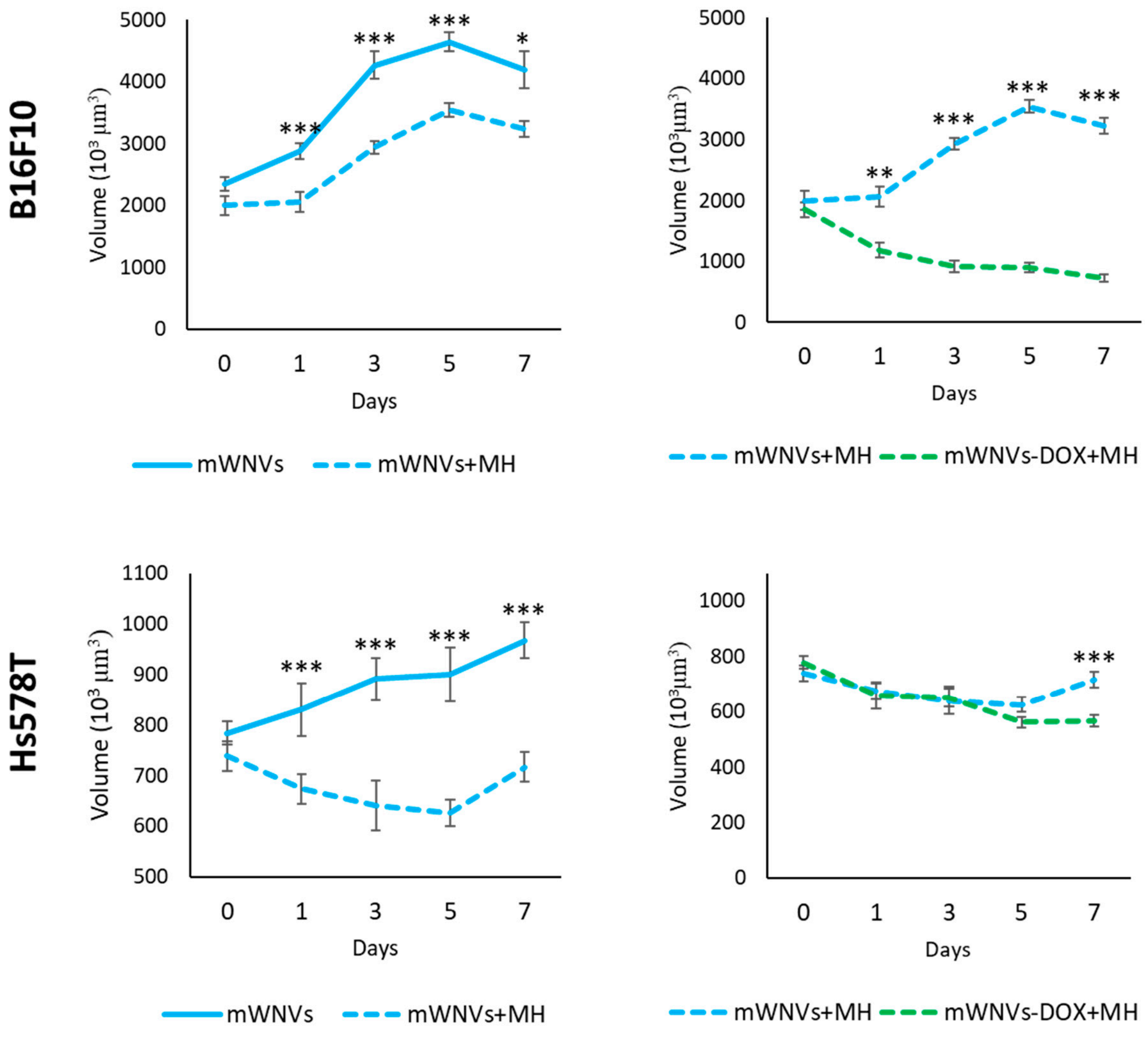

Figure S16. Volume evolution of B16F10 MCTSs (top) and Hs578T MCTSs (bottom) treated with control unloaded mWNVs (blue) and mWNV-DOX (green), alone (solid lines) and under 1 h magnetic hyperthermia application (dotted lines) measured for 7 days (mean ± SEM; * $p < 0.01$, ** $p < 0.005$, *** $p < 0.001$., Student *t* test).

Table S1. Criteira followed for the designation of the effect of the combination thermo- chemo-therapy.

**Effect**	**Formula** [1]
**Synergistic**	$(A + B) < \frac{A \times B}{100}$
**Additive**	$(A + B) = \frac{A \times B}{100}$
**Sub-additive**	$\frac{A \times B}{100} < (A + B) < A; if\ A < B$
**Interference**	$A < (A + B) < B, if\ A < B$
**Antagonistic**	$B < (A + B), if\ A < B$

MTCS

For B16F10:

A (mWNVs-DOX) = 33.44% final volume

B (mWNVs) = 85.11% final volume

(A+B) (mWNVs-DOX + MH) = 19.16% final volume

Synergy: (A+B) < (A x B)/100 ⇨ 19.16 < 28.46

For Hs578T:

A (mWNVs-DOX) = 82.65% final volume

B (mWNVs) = 73.47% final volume

(A+B) (mWNVs-DOX + MH) = 58.16% final volume

Synergy: (A+B) < (A xB)/100⇨ 58.16 < 60.72

Reference

1. Pradhan, P.; Giri, J.; Rieken, F.; Koch, C.; Mykhaylyk, O.; Döblinger, M.; Banerjee, R.; Bahadur, D.; Plank, C. Targeted temperature sensitive magnetic liposomes for thermo-chemotherapy. *J. Control. Release* **2010**, *142*, 108–121.
